# Ten tips for kidney biopsy in cancer patients

**DOI:** 10.1093/ckj/sfag218

**Published:** 2026-08-05

**Authors:** Mariadelina Simeoni, Mario Ollero, Clara Garcia-Carro, Corinne Bagnis, Andreas Kousios, Gerrit van den Berg, Maria Jose Soler, Vladimir Tesar, Jolanta Malyszko

**Affiliations:** Department of Translational Medical Sciences, University of Campania Luigi Vanvitelli, Naples, Italy; Department of Nephrology, Dialysis and Kidney Transplant Follow-up, Luigi Vanvitelli University Hospital Naples, Italy; Paris Est Creteil University, Créteil, France; Mondor Institute (INSERM U955),Institute Mondor de Recherche Biomédicale, Créteil, France; Nephrology Department, San Carlos University Clinical Hospital, IdSSC, Madrid, Spain; Madrid Complutense University, Madrid, Spain; Nephrology Department, AP-HP University, Paris, France; Nefronida Medical Centre, Nicosia, Cyprus; School of Medicine, European University of Cyprus, Nicosia, Cyprus; Princess Máxima Center for Pediatric Oncology, Utrecht, The Netherlands; Department of Pediatric Nephrology, Wilhelmina Children’s Hospital/University Medical Center Utrecht, Utrecht, The Netherlands; Department of Nephrology, Vall Hebrón University Hospital, Barcelona, Spain; Department of Nephrology, First School of Medicine and General University Hospital, Charles University, Prague, Czech Republic; Department of Nephrology, Dialysis and Internal Medicine, Medical University of Warsaw, Warsaw, Poland

**Keywords:** kidney diseases, kidney transplantation, neoplasms, nephrotoxicity, paediatric oncology, renal biopsy, thrombotic microangiopathies

## Abstract

Renal biopsy is an essential diagnostic tool to be considered in cancer patients, a population in whom renal dysfunction is frequent, multifactorial, and clinically significant. Accurate identification of the underlying lesion is often crucial for guiding oncologic therapy, preventing further renal decline, and improving overall outcomes. However, performing a biopsy in this vulnerable patient group requires careful evaluation of procedural risks and clinical context.

This ‘10 Tips’ paper provides a practical framework to support clinicians in selecting appropriate candidates, preparing for the procedure, and interpreting histopathological findings. Special attention is given to high-risk and end-of-life scenarios, where the balance between diagnostic benefit and potential harm becomes particularly delicate. By integrating clinical, procedural, and ethical considerations, this paper aims to promote thoughtful, patient-centred approach to renal biopsy in the complex landscape of onconephrology. Shared decision-making and multidisciplinary collaboration among nephrologists, oncologists, haematologists, and pathologists are essential.

## Graphical Abstract

**Graphical Abstract:**
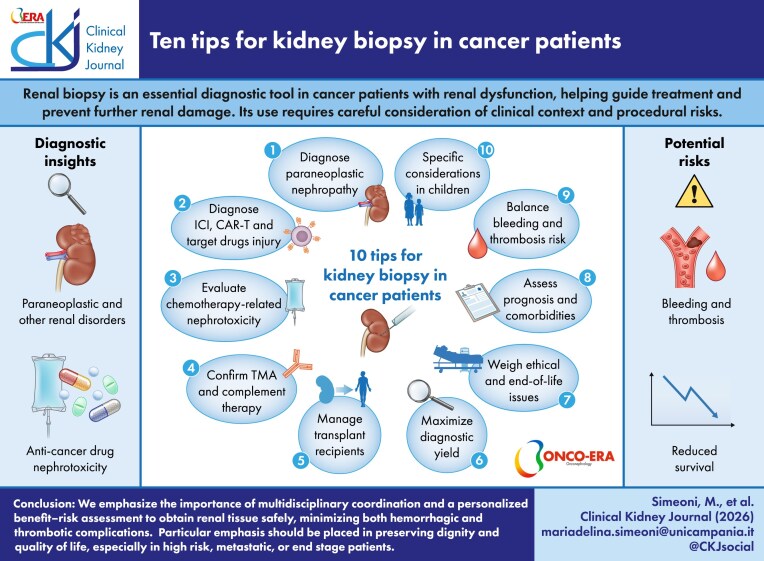

## INTRODUCTION

Cancer survival rates have substantially improved with the advent of innovative therapies; however, patients with cancer and concomitant renal dysfunction continue to experience poorer outcomes and higher mortality compared with those with preserved kidney function [1]. This disparity underscores the need for a personalized and pathophysiology-targeted approach to the assessment and management of renal disease in patients with cancer.

Current recommendations for renal biopsy in these patients are outdated, with the most recent guidance originating from the 2020 Kidney Disease: Improving Global Outcomes (KDIGO) Consensus. In this document, renal biopsy is recommended for patients with cancer who present with new-onset proteinuria >1 g/day or worsening renal function when the diagnosis of kidney disease cannot otherwise be established. In addition, the indication for renal biopsy in patients with cancer and favourable oncologic outcomes or expected long-term survival should be similar to that in the general population [2, 3].

We provide 10 practical tips to guide the nephrology community in deciding whether to perform renal biopsy. The role and clinical utility of kidney biopsy within the clinical context of various cancer-related scenarios will be discussed. These include paraneoplastic glomerular diseases, nephrotoxicity associated with different classes of anticancer therapies, oncohaematological disorders with renal involvement, cancer in kidney transplant recipients (KTRs), paediatric patients, and individuals with end-stage malignancy. A special focus on the delicate thrombotic/haemorrhagic risk and on ethical aspects of renal biopsy in cancer patients will also be addressed.

## TEN TIPS ON RENAL BIOPSY IN CANCER PATIENTS

Renal biopsy is a pivotal yet often underutilized diagnostic tool in cancer patients with renal dysfunction. Since the 2020 KDIGO Onco-Nephrology Controversies Conference first highlighted the unmet need for guidance on this topic, accumulating real-world evidence has reinforced the diagnostic value and acceptable safety profile of renal biopsy in selected oncologic populations [2]. Recent observational cohorts have shown that biopsy frequently leads to diagnostic reclassification and meaningful changes in management, particularly in the context of immunotherapy and targeted therapies-related kidney injury, thrombotic microangiopathy (TMA), and oncohaematologic diseases [4, 5].

At the same time, advances in supportive care and procedural techniques have improved biopsy safety even in complex cancer settings, provided that careful patient selection is applied. Alongside histology, noninvasive approaches, including urinary and circulating biomarkers, molecular profiling, and functional or metabolic imaging, are emerging as complementary tools to refine patient selection and, in selected scenarios, potentially reduce the need for invasive procedures. However, until such strategies are prospectively validated, renal biopsy remains central to diagnostic precision and personalized decision-making in onconephrology [6].

In general, renal biopsy should be considered in the presence of unexplained, atypical, or rapidly progressive kidney abnormalities, or when histological information is likely to influence the therapeutic pathway. Conversely, deferral should be pursued when biopsy is unlikely to provide actionable information or when the procedure is unsafe or ethically unjustifiable. Table 1 summarizes the typical indications and contraindications across different clinical scenarios, considering risk–benefit balance and ethical considerations, according to the 10 practical recommendations proposed by the ONCO-ERA Working Group and discussed below.

**Table 1:** ONCO-ERA Working Group recommendations on renal biopsy versus deferral in cancer patients with renal dysfunction, with indications organized into individual practical tips.

TEN TIPS	PERFORM renal biopsy	DEFER renal biopsy
*1. Pre*-*existing CKD vs paraneoplastic renal disease*	Unexplained or abrupt change in previously stable CKD Nephrotic range proteinuria Haematuria Renal dysfunction synchronous with cancer diagnosis or relapse	Stable, well-documented CKD with unchanged course Renal impairment fully explained by diabetes, hypertension, or chronic noncancer**-**related disease
*2. ICIs/CAR*-*T therapies*	Moderate–severe or persistent unexplained AKI Haematuria or significant proteinuria Need to distinguish immune mediated injury from other causes to guide therapy	Mild, rapidly reversible AKI; clear alternative cause Clinical instability or cytopenias precluding safe biopsy (especially CAR-T setting)
*3. Conventional chemotherapy*	Persistent AKI despite drug withdrawal and supportive hydration New-onset, rapidly progressive, or nephrotic range proteinuria Haematuria and/or urinary casts When histology may influence continuation or change of therapy	AKI clearly linked to recent chemotherapy with expected reversibility Purely haemodynamic or obstructive causes
*4. Targeted therapies (anti*-*VEGF and TKIs)*	Persistent proteinuria or AKI despite drug discontinuation Suspected TMA	Mild, reversible hypertension or proteinuria AKI resolving after therapy withdrawal Pseudo-AKI
5. *Suspected TMA*	Suspected cancer-associated TMA Unexplained AKI with anemia and thrombocytopenia Suspected complement-mediated TMA/aHUS HSCT-associated TMA	Advanced irreversible kidney damage Clear clinical diagnosis of TMA High procedural bleeding/risk
*6. Oncohaematological renal dysfunction*	Unclear renal etiology Suspected MGRS, AL amyloidosis, or lymphomatous infiltration Free light chains <500 mg/l Significant albuminuria or atypical presentation	Suspected MCN with very high free light chains (>500 mg/l) requiring immediate treatment Advanced kidney damage
*7. Paediatric cancer patients*	Atypical or unexplained renal dysfunction suggesting alternative diagnoses (e.g. KIN after ifosfamide)	Renal injury clearly attributable to known chemotherapy toxicity with typical clinical course
*8. KTRs with cancer*	Unexplained graft dysfunction Suspected rejection, drug toxicity, recurrence of original disease, or tumour infiltration	Advanced graft failure unlikely to benefit from diagnostic clarification
*9. Anticoagulation/bleeding risk*	Acceptable bleeding risk after multidisciplinary assessment and optimization	Uncorrectable coagulopathy Severe thrombocytopenia Inability to safely interrupt anticoagulation
*10. Ethical/end*-*of*-*life considerations*	Expected impact on management aligned with patient goals of care	Limited life expectancy Advanced metastatic disease If results will not change the management

### Differentiation of pre-existing renal disease from paraneoplastic renal manifestations

Renal disorders in patients presenting with a malignancy may arise either from pre-existing chronic kidney disease (CKD) or from paraneoplastic mechanisms [3, 7–9].

Because cancer-related and noncancer-related kidney diseases frequently present with overlapping clinical features such as proteinuria, nephrotic syndrome, haematuria, and progressive decline in kidney function, precise characterization of the causative histopathological process is essential. This differentiation is further complicated by the high prevalence of common causes of CKD, including hypertension and diabetes, in patients with cancer [10].

A key element in distinguishing pre-existing kidney disease from paraneoplastic renal syndromes is careful evaluation of the patient’s renal history and its longitudinal course, as documented through prior clinical assessments and laboratory findings. Any unexpected change during routine follow-up should prompt consideration of a superimposed pathological process. Typical examples include patients with long-standing diabetes and/or hypertension, who develop new-onset nephrotic-range proteinuria, rapidly progressive glomerulonephritis or vasculitis, or TMA. When such changes occur, particularly in the presence of malignancy risk factors, an underlying neoplastic process should be actively investigated. Similarly, a strong family history of cancer should raise suspicion for an occult malignancy when the clinical course of kidney disease becomes atypical [11].

In this context, the decision to perform a renal biopsy in patients with cancer should be guided by well-defined clinical and biochemical criteria and in a multidisciplinary setting. Primary indications include unexplained or atypical proteinuria and rapidly progressive or otherwise unexplained deterioration of renal function [2].

Furthermore, a biopsy is strongly warranted when renal manifestations recur in temporal association with tumour relapse, as this pattern may indicate an indirect paraneoplastic or infiltrative process. In these settings, histological evaluation is essential to differentiate irreversible chronic renal lesions from potentially treatable malignancy-related renal injury. Renal biopsy can also reveal characteristic pathological patterns supporting a paraneoplastic etiology, such as membranous nephropathy associated with the positivity of specific antibodies which have been increasingly linked to underlying solid tumours (e.g. anti-thrombospondin type-1 domain-containing 7A or antineural epidermal growth factor-like 1 protein) [12]. Other examples are those of minimal change disease concomitant to different lymphomas [7] and selected TMA or proliferative glomerulopathies consistent with paraneoplastic syndromes [13]. Moreover, differences in the pattern, composition, and distribution of immune-complex deposits, often more heterogeneous, atypical, or accompanied by tumour-related antigens in paraneoplastic nephropathy, can help distinguish it from the more stereotyped and disease-specific deposition patterns seen in primary glomerulonephritis [14]. In addition, immunoglobulin subclass distribution may differ and is often skewed or atypical in malignancy-associated glomerulopathies [10, 15]. These lesions also show more heterogeneous or tumour-driven inflammatory cell infiltrates. In contrast, primary or idiopathic forms typically display stereotyped subclass patterns and disease-specific inflammatory profiles [11, 16, 17].

### Diagnosis of immune-related kidney injury from checkpoint inhibitors and Chimeric Antigen Receptor (CAR) -T cell therapy.

In patients receiving immune checkpoint inhibitors (ICIs), the development of acute kidney injury (AKI) is mostly a multifactorial challenge in which the role of renal biopsy has become increasingly central. Most contemporary cohorts confirm that acute tubulointerstitial nephritis (ATIN) is the dominant lesion found in ICI-AKI [18]. Conversely, glomerular diseases such as pauci-immune vasculitis, podocytopathies, or C3 glomerulopathy, are distinctly uncommon but crucial to identify given their therapeutic implications [19]. For this reason, renal biopsy is particularly important in patients who present with moderate-to-severe AKI, or in cases of persistent, unexplained stage 1 AKI. Likewise, the appearance of haematuria, significant proteinuria, or other features suggestive of renal dysfunction strongly reinforce the need for histological evaluation. Recently published expert consensus statements highlighted that biopsy is often the only way to avoid unnecessary corticosteroid exposure [20], and to prevent inappropriate interruption of immunotherapy [21].

Once confirmed, ATIN typically shows dense lymphocytic interstitial inflammation with plasma cells and active tubulitis, and biopsy-guided management allows for shorter, individualized steroid regimens without higher recurrence risk [1, 22]. Emerging biomarkers, including imaging signatures such as Fluorodeoxyglucose Positron Emission Tomography (FDG-PET) renal uptake and urinary cytokine or chemokine profiles, hold promise as future tools to noninvasively diagnose immune-mediated nephritis [23]. Although still under investigation, these markers could ultimately complement or even replace biopsy in selected cases.

AKI following CAR-T cell therapy is common but typically mild and reversible because it is linked to systemic inflammation rather than to direct renal immune toxicity [24]. Proteinuria is frequent, but predominantly tubular, and transient, often peaking around day +7 in parallel with inflammatory cytokine release [25]. Given cytopenias and clinical instability, renal biopsy is rarely feasible in this setting, and its role remains limited.

### To investigate AKI in cancer patients undergoing conventional chemotherapy

Renal toxicity is a well-recognized limitation of many cytotoxic chemotherapeutic agents and represents a major challenge in the management of patients with solid and haematologic malignancies [26, 27]. Clinical manifestations, including AKI, proteinuria, electrolyte disturbances, and rapid transition to CKD are common but often nonspecific. The underlying histopathological patterns of injury are highly heterogeneous and cannot be reliably inferred from clinical or laboratory data alone. In this context, renal biopsy remains a useful diagnostic tool to elucidate the mechanisms of chemotherapy-related nephrotoxicity and to guide individualized therapeutic decisions. Cytotoxic agents such as platinum compounds, antimetabolites, alkylating agents, and other targeted cytotoxic drugs can induce a broad spectrum of renal lesions. These include acute tubular necrosis, ATIN, TMA various glomerulopathies, and chronic interstitial fibrosis [28–31]. Biopsy is particularly indicated in unexplained or severe AKI, especially in the presence of multiple nephrotoxic exposures, relevant comorbidities, or an uncertain temporal association with chemotherapy.

New-onset or rapidly progressive proteinuria, especially in the nephrotic range, strongly supports the need for histological clarification, as does the presence of active urinary sediment with haematuria and/or casts [32]. Finally, biopsy should be pursued when histological information is expected to directly influence oncologic decision-making, such as determining whether continuation of a key chemotherapeutic agent is feasible with nephroprotective strategies [32, 33], or whether a switch to a less nephrotoxic regimen is required. [34–36].

### Diagnosis of kidney injury from targeted therapies

Renal toxicity is a well-recognized complication of therapies targeting vascular endothelial growth factor (VEGF) or its receptor-associated tyrosine kinases. It most commonly presents as new-onset or worsening hypertension and/or proteinuria. The latter has been reported in one-fifth to nearly two-thirds of treated patients. Only a small proportion, generally between 2% and 6%, develop proteinuria of nephrotic severity [37]. In addition, a subset of individuals may experience a measurable decline in kidney function, further underscoring the increased susceptibility to renal injury associated with anti-VEGF therapies [38]. Because renal biopsy is infrequently—performed in this clinical context, the true incidence and long-term consequences of anti-VEGF inhibitors-associated nephrotoxicity are likely underestimated.

This concern is supported by data from larger cohorts. In a series of 100 patients with kidney disease attributed to anti-VEGF therapy, the spectrum of renal injury was broad and often clinically significant, underscoring the need for systematic evaluation and long-term follow-up [38]. TMA was the predominant lesion, identified in 73% of cases, whereas podocytopathies were less common. The latter included minimal change disease and focal segmental glomerulosclerosis (FSGS), most often presenting as the collapsing variant, which together accounted for 27% of patients. Discontinuation of anti-VEGF therapy, together with appropriate antihypertensive treatment, led to improvement of both hypertension and proteinuria in most patients, and none progressed to kidney failure requiring dialysis. However, among those who continued anti-VEGF therapy, recurrence of TMA occurred in three of the four patients who remained on treatment [39]. Similar patterns of nephrotoxicity, characterized by hypertension, proteinuria, and episodes of AKI, often accompanied by clinicopathological evidence of TMA have also been reported in patients receiving the anti-VEGF monoclonal antibody bevacizumab. In most cases, these abnormalities improved after discontinuation of therapy, although reversibility was not universal [40].

A recent small series of biopsied patients with renal toxicity related to tyrosine kinase inhibitors (TKIs) has confirmed that TMA is observed more frequently than FSGS, including cases in which both lesions coexist, in individuals treated with anti-VEGF agents. Comparable patterns have also been described in patients receiving multikinase inhibitors that additionally target the platelet-derived growth factor receptor-associated tyrosine kinase. Despite the addition of corticosteroids to an anti-Renin-Angiotensin-Aldosterone System (RAAS) agent following discontinuation of the offending agent, nephrotoxicity still recurred in approximately half of the treated patients [41]. Another series of 22 biopsied patients with nephrotoxicity related to anti-VEGF therapy (including bevacizumab, lenvatinib, and sorafenib) showed a clinical presentation dominated by hypertension and non-nephrotic proteinuria, with TMA identified in most cases (86%). AKI resolved within several weeks in a large part of patients [42]. A recent consensus paper underlined the importance of kidney health assessment before starting any potentially nephrotoxic anticancer therapy and, as hypertension occurs in 30%–80% of patients treated with anti-VEGF therapies, regular measurement of blood pressure and antihypertensive treatment optimization is warranted. Discontinuation of anti-VEGF agents is immediately necessary in nephrotic patients and/or patients with AKI and TMA [43]. Renal biopsy is not routinely recommended in all patients with anti-VEGF-associated nephrotoxicity, but should be considered in patients with persistent renal symptoms despite withdrawal of treatment.

### TMA characterization guiding the use of anticomplement agents in appropriate patients

TMA encompasses a spectrum of disorders characterized by endothelial injury, microvascular thrombosis, and organ dysfunction, particularly affecting the kidneys [44–46]. Cancer-associated TMA is a rare but serious complication that may arise from the malignancy itself or because of chemotherapy, targeted therapies, ICIs, CAR-T cell therapy, or haematopoietic stem cell transplantation (HSCT) [47]. Agents such as gemcitabine, mitomycin C, and anti-VEGF therapies are known to induce endothelial injury, leading to microvascular thrombosis and renal dysfunction [48]. Within ICIs, atezolizumab, nivolumab, and pembrolizumab have been implicated in TMA development [49].

Haematologic malignancies, especially acute leukaemias and lymphomas, are most associated with TMA, followed by advanced solid tumours such as gastric, breast, and prostate cancers [49]. Patients may present with anemia, thrombocytopenia, and AKI, all symptoms that overlap with other oncologic complications making TMA diagnosis even more challenging. Renal biopsy provides critical histological evidence, revealing features like fibrin thrombi in glomeruli and arterioles, endothelial swelling, and mesangiolysis, which confirm TMA and help differentiate it from other causes of renal dysfunction [50]. Importantly, immunofluorescence can help identify complement-mediated TMA such as atypical haemolytic uremic syndrome (aHUS), which may occur independently or be triggered by cancer therapies [51].

In such cases, (e.g. stem cell transplantation-associated TMA), anti-C5b agents like eculizumab or ravulizumab may be considered, especially if the renal biopsy shows active endothelial injury with minimal chronic damage [52]. This is crucial because complement inhibitors are expensive and carry immunosuppressive risks which may expose cancer patients to disease progression. Moreover, biopsy can reveal concurrent pathologies, like superimposed thrombotic lesions on pre-existing glomerulopathies, which may alter therapeutic strategies [53]. Conversely, when a clear clinical diagnosis is present in the setting of advanced, irreversible kidney damage or an unacceptably high procedural risk, renal biopsy should be deferred in patients with suspected TMA (Table 1).

### Guide to treatment decisions in oncohaematologic patients with renal dysfunction

Among all cancer types, haematological malignancies exhibit the highest rates of kidney injury, with multiple myeloma (MM) and leukaemias accounting for most malignancy-related renal diseases [29]. Kidney dysfunction occurs at presentation or during the disease course in ∼10%–30% of patients with lymphomas and leukaemias, increasing to 60%–70% in multiple myeloma (MM) and light chain-amyloidosis (AL), HSCT recipients [54, 55]. Pathology arises through diverse mechanisms, including paraprotein-mediated injury, tumour infiltration, paraneoplastic glomerulopathies, metabolic disturbances, tumour lysis syndrome, and treatment-related nephrotoxicity. Kidney impairment is associated with increased morbidity and mortality and may limit therapeutic options, including eligibility for intensive chemotherapy or transplantation [56]. Definitive diagnosis often requires a renal biopsy, although clinicians may hesitate due to perceived procedural risks. Early recognition and timely histological assessment are essential to guide targeted therapy and improve outcomes.

In MM, renal impairment accompanied by serum free light chain concentrations >500 mg/l is highly predictive of myeloma cast nephropathy (MCN), warranting immediate antimyeloma therapy without delaying for a renal biopsy. In this setting, the speed of haematologic response is a key determinant of kidney recovery and long-term kidney survival [57]. Nonetheless, even in MCN, a growing body of evidence, suggests that renal biopsy findings can have added diagnostic value, uncovering concomitant pathology, such as monoclonal immunoglobulin deposit disease (MIDD) or AL amyloidosis, and offer important prognostic information [58, 59]. In patients with free light chains <500 mg/l and/or marked albuminuria, without typical signs of myeloma, renal biopsy is recommended to differentiate AL amyloidosis, MIDD, or other paraprotein-related renal diseases, guiding treatment.

Further diagnostic work-up and treatment strategies can then be tailored according to the identified renal disease [60].

Monoclonal gammopathy of renal significance (MGRS) comprises kidney lesions caused by nephrotoxic monoclonal proteins arising from low-grade B-cell or plasma-cell clones that do not meet criteria for symptomatic lymphoma or myeloma [61]. Renal biopsy is essential for establishing the diagnosis and for distinguishing MGRS from monoclonal gammopathy of undetermined significance. It can uncover a wide spectrum of renal lesions, ranging from subtle proximal tubulopathies to podocytopathies, proliferative glomerulonephritis with monoclonal immunoglobulin deposits (PGNMID), cryoglobulinaemic glomerulopathy, and complement-mediated diseases, among others [62].

Notably, without accurate diagnosis and clone-directed therapy, MGRS typically progresses to end-stage kidney disease. In lymphoproliferative disorders, renal biopsy may reveal not only MGRS lesions but also paraneoplastic processes such as minimal change disease or TMA as well as direct lymphomatous infiltration. The latter is often underrecognized owing to its subtle presentation. Early biopsy is crucial, as timely lymphoma-directed therapy can lead to rapid renal recovery [54, 63]. Kidney injury is common after HSCT (30%–70%) and markedly increases mortality. Etiologies are diverse (conditioning regimens, irradiation, nephrotoxins, infection, transplantation-associated TMA, and graft-versus-host disease), and can be definitively distinguished only by renal biopsy. Early identification of the specific lesion is essential to guide targeted therapy and improve outcomes [55].

### Limited application of renal biopsy in children with cancer and renal dysfunction

In children, there is always a degree of caution regarding kidney biopsies due to the risks of the procedure. General anaesthesia or sedation is required, and the smaller kidney size makes the procedure technically more challenging, although percutaneous renal biopsy is also a low-risk procedure in this age group [64]. Nonetheless, the procedure is less commonly performed, and renal biopsy is seldom reported in the context of kidney complications secondary to cancer. Given the low prevalence of CKD in childhood [65], renal biopsy to differentiate pre-existing kidney pathology from paraneoplastic lesions is generally not warranted. Nevertheless, proteinuria may occur as a paraneoplastic feature. The evidence supporting an association between nephrotic syndrome and haematologic malignancies is limited. Reported cases primarily involve paediatric patients with Hodgkin lymphoma, with an estimated incidence of up to 1%. Case reports suggest that nephrotic syndrome may resolve following initiation of chemotherapy [66]. Regarding kidney injury related to ICIs, anti-VEGF, and CAR-T cell therapy, reports in children are very scarce [67], and the considerations established in adults appear to be generally applicable until paediatric-specific literature becomes available [68].

With respect to conventional chemotherapy, ifosfamide [69] and methotrexate [70] are considered major risk factors for AKI. In addition, ifosfamide, cisplatin, carboplatin, kidney-exposing radiotherapy (KER), nephrectomy, and total body irradiation (TBI) have been identified as risk factors for decreased renal function, whereas ifosfamide, TBI, and KER are associated with an increased risk of proteinuria [71]. In such treatment-related contexts, kidney impairment is typically attributable to therapy-induced nephrotoxicity, and renal biopsy is generally not recommended unless clinical features suggest an alternative diagnosis. A notable differential diagnosis in this setting is karyomegalic interstitial nephropathy (KIN). Following ifosfamide therapy, some children may develop rapidly progressive renal dysfunction, and case series have identified KIN as a potential cause. KIN is characterized by markedly enlarged nuclei in tubular epithelial cells and represents a distinct clinicopathological entity in children with CKD after exposure to ifosfamide. Early recognition is important, as timely diagnosis may guide management. Limited evidence suggests a possible preventive effect of corticosteroids; however, no specific treatment has been established so far [72].

### Renal pathology assessment in kidney transplant recipients with cancer

KTRs have a 2–4-fold higher risk of malignancy compared with the general population, and cancer represents a major cause of death alongside cardiovascular disease and infection [73]. In addition to cancer types common in the general population, KTRs are particularly susceptible to transplant-associated malignancies, including nonmelanoma skin cancers [74], post-transplant lymphoproliferative disorders, Kaposi sarcoma, and native kidney renal cell carcinoma [75].

Multiple processes can impair allograft function in KTRs with cancer: acute rejection, chronic alloimmune injury, anticancer drugs-related nephrotoxicity, direct cancerous infiltration, or paraneoplastic glomerular disease. These entities require different treatments but are most of the time indistinguishable without histological evaluation. Worsening graft function or allograft failure restricts cancer treatment options and is associated with increased morbidity and mortality in a population with limited access to re-transplantation. Immunosuppression is often reduced after cancer diagnosis, typically by lowering tacrolimus exposure, tapering steroids, and discontinuing antimetabolites, which increase the risk of acute rejection and graft failure. The early identification of alloimmune graft injury is therefore essential to preserve graft survival [76]. Conventional cancer treatment can be directly nephrotoxic in the kidney graft causing tubular injury (e.g. cisplatin, ifosfamide, and high-dose methotrexate) or TMA (e.g. gemcitabine) [77].

ICIs have emerged as a promising cancer therapy in KTRs, with effective anticancer responses achievable in a subset of patients. However, only about one-third attain disease control while retaining graft function, with case series reporting rejection rates of 40%–50% [78]. Allograft rejection typically occurs early after ICIs initiation and is frequently associated with irreversible graft loss if not promptly recognized and treated. Although emerging noninvasive biomarkers such as donor-derived cell-free DNA and urinary chemokine C–X–C motif ligand (CXCL10) may allow earlier detection of alloimmune injury than serum creatinine, kidney allograft biopsy remains the definitive diagnostic tool to guide immunosuppression adjustment, determine the need for antirejection therapy versus ICIs discontinuation, and balance graft preservation with effective cancer control [79, 80].

Certain haematological malignancies are prone to relapse after kidney transplantation, and early post-transplant renal biopsy is critical for timely diagnosis and improved outcomes. In PGNMID, recurrence rates are exceptionally high, approaching 90% within a few months after transplantation, with detection significantly enhanced by protocol biopsies [81]. In contrast, recurrence of AL amyloidosis in the kidney allograft typically occurs later, at a median of ∼6.6 years post transplant, and timely clone-directed haematological therapy can prevent graft loss in most patients [82].

### Renal biopsy in the light of the delicate thrombotic/haemorrhagic risk balance in cancer patients

Renal biopsy in cancer patients often takes place in a context of increased haemorrhagic and thrombotic risk due to active malignancy, recent oncologic procedures, thrombocytopenia, or treatment with antiplatelet or anticoagulant agents. In large contemporary series of native kidney biopsies, the overall rate of major complications ranges from ∼2% to 5%, depending on definitions and populations, with clinically relevant outcomes including transfusion, embolization, and rare mortality [83]. A national registry analysis reported major complications in ∼2.1% of native biopsies, with embolization in ∼0.63%, transfusion-only in ∼0.34%, and biopsy-attributable mortality ∼0.15%. A large prospective multicentre study reported an ∼5.1% major complication rate (including ≥2 g/dl haemoglobin drop 2.2%, transfusion 1.1%, invasive intervention 0.5%, and death 0.02%). While these data derive from mixed (nononcology-specific) populations, they provide a practical benchmark for counselling. The cancer setting may increase both competing thrombotic risk and procedural complexity, reinforcing the need for individualized decision-making [83, 84].

Multiple studies show that bleeding risk in not increased in monoclonal gammopathies, although traditionally considered at high risk [85]. No evidence is available in other cancer contexts. Recent imaging is recommended to rule out anatomical contraindications or features requiring special precautions (single kidney, renal atrophy, cysts, tumour, hydronephrosis, and graft-specific risks). Spontaneous or procedure-related bleeding should be systematically documented. Prothrombin Time (PT) and Activated Partial Thromboplastin Time (aPTT) should be measured in case of patients with specific comorbidities or a history suggesting a haemostasis disorder; otherwise, routine haemostasis testing is not mandatory but should be justified in the medical record. Platelets count <50 × 109/l or haemoglobin <8 g/dl represent absolute contraindications to renal biopsy performing, while thresholds between 50 and 100 × 10/l (platelets) or 8 and 10 g/dl (Hb) require individualized benefit–risk assessment. Bleeding time and platelet occlusion time are not recommended. When abnormalities are detected, haematology consultation is advised. Desmopressin should not be used unless specifically indicated [86, 87].

In order to manage eventual ongoing antiaggregant/anticoagulant therapies before renal biopsy, the involvement of the prescribing specialist and/or the advice of a haematologist is essential. Aspirin may be continued when the risk of thrombosis outweighs bleeding risk; if interrupted, it should be stopped 5 days before the biopsy and restarted after 24 hours in the absence of complications. Clopidogrel, prasugrel, and ticagrelor should be withheld (5–7 days depending on the agent). A temporary switch to low-dose aspirin may be considered, but it is not mandatory [88]. For patients on a vitamin K antagonist, INR should be checked 7 days before the biopsy, with discontinuation planned to reach International Normalized Ratio (INR) <1.5 on the day of the procedure. Bridging with heparin is reserved for high-risk thromboembolic situations (mechanical valves, recent venous thromboembolism, antiphospholipid syndrome, and heparin-induced thrombocytopenia) [89].

**Figure 1:**
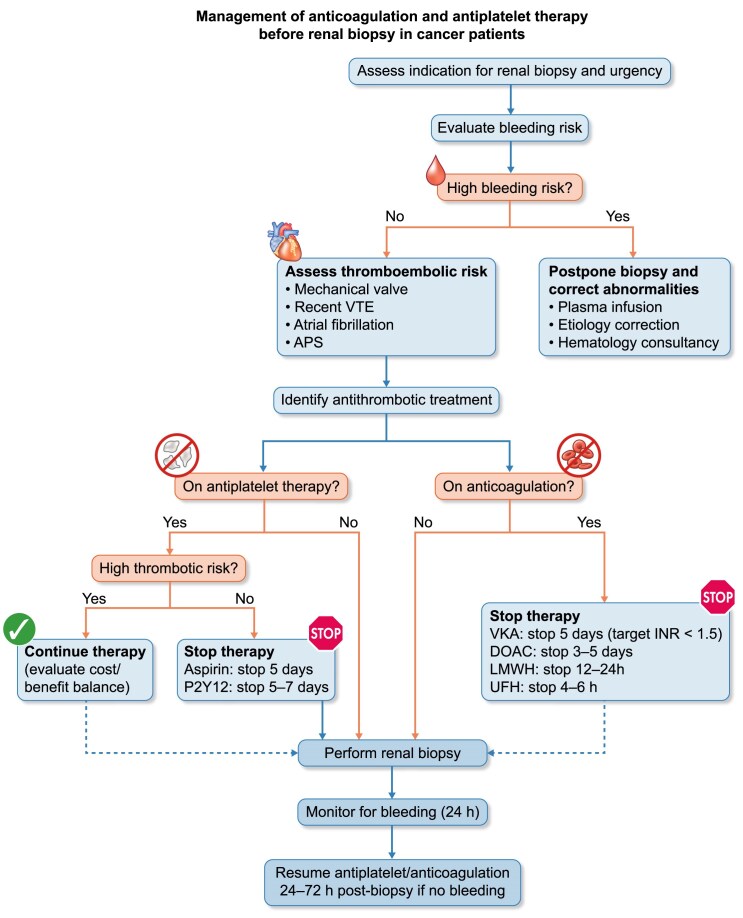
Management of anticoagulation and antiplatelet therapy before renal biopsy in cancer patients. Algorithm outlining the evaluation of bleeding and thromboembolic risk prior to renal biopsy, including identification of ongoing antithrombotic therapy, assessment of biopsy urgency, and decisions regarding continuation or temporary discontinuation of anticoagulant or antiplatelet agents. The figure summarizes recommended interruption intervals, supportive measures for high bleeding risk, and post-biopsy monitoring and timing for therapy resumption.

Low molecular weight heparin should be stopped 24 hours before the procedure, and unfractionated heparin 6 hours before the biopsy. Direct oral anticoagulants should be discontinued 3–5 days before renal biopsy depending on the molecule and renal function. Anticoagulant resumption generally occurs 24–72 hours post biopsy in the absence of bleeding [90]. In Fig. 1, an algorithm is presented illustrating the evaluation of bleeding and thromboembolic risk before renal biopsy. The workflow includes identification of ongoing antithrombotic therapy, assessment of biopsy urgency, and guidance on continuation or temporary interruption of anticoagulant and antiplatelet agents. The algorithm also summarizes recommended drug interruption intervals, supportive measures for patients at high bleeding risk, and postbiopsy monitoring with timing for therapy resumption.

### Ethical considerations on renal biopsy performing in high-risk or end-of-life scenarios

Renal biopsy in oncological patients presents a complex set of ethical challenges, especially in high-risk or end-of-life situations where the balance between potential benefit and harm becomes particularly delicate. The ethical imperative of nonmaleficence is mandatory when the likelihood of altering clinical management is low [91].

In these scenarios, autonomy requires robust, transparent communication: patients must understand not only procedural risks but also whether biopsy results would meaningfully influence therapeutic decisions or confirm a clinical suspicion without changing the trajectory of care [92]. Literature on end-of-life oncology emphasizes the importance of aligning interventions with patient-centred goals, cautioning against ‘diagnostic momentum’ that may shift focus away from comfort-oriented care [93]. Ethical frameworks also highlight justice, particularly regarding the use of invasive diagnostics with limited expected benefit in patients nearing the end of life. Guidelines in renal cancer management acknowledge the role of biopsy but stress individualized assessment, especially when frailty or advanced disease limits the utility of further diagnostic escalation [94].

Frailty can be rapidly stratified with the Clinical Frailty Scale [95] functional reserve and treatment tolerance can be contextualized using Eastern Cooperative Oncology Group (ECOG) Performance Status [96]. Short-term prognosis and communication about time-limited benefit can be supported by the Palliative Performance Scale [97]. Ultimately, ethical decision-making around renal biopsy in oncological patients requires interdisciplinary dialogue, careful prognostic consideration, and a commitment to preserving dignity and quality of life. The latter represents a fundamental outcome, particularly crucial in the care of patients with end-stage conditions and those living with cancer, in whom personality traits and coping strategies play a central role and may influence decisions regarding invasive procedures and treatment options [98].

## CONCLUSIONS

**Figure 2:**
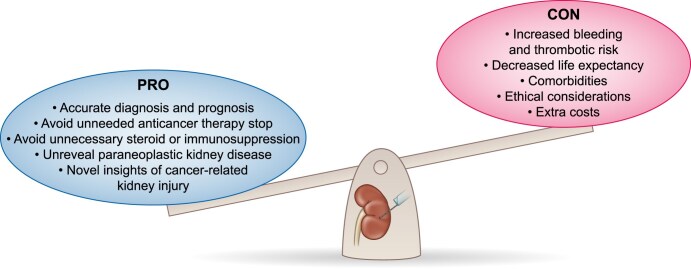
Risk–benefit assessment of renal biopsy in cancer patients. Renal biopsy can refine diagnosis, guide prognosis, prevent unnecessary treatment changes, and uncover paraneoplastic kidney disease, but must be balanced against bleeding and thrombotic risks, comorbidities, limited life expectancy, ethical concerns, and added costs.

Renal dysfunction in cancer patients may arise from paraneoplastic manifestations, direct neoplastic infiltration of the kidney, or nephrotoxicity related to anticancer therapies. This population represents a uniquely complex patient group in which the decision to perform a renal biopsy must balance diagnostic accuracy with the need for individualized therapeutic strategies (Fig. 2). The ONCO-ERA Working Group emphasizes the importance of multidisciplinary coordination and a personalized benefit–risk assessment to obtain renal tissue safely, minimizing both haemorrhagic and thrombotic complications. Particular emphasis should be placed in preserving dignity and quality of life, especially in high-risk, metastatic, or end-stage patients, in whom diagnostic and therapeutic obstinacy may entail significant ethical and legal implications.

## Data Availability

No new data were generated or analyzed in support of this research.
